# Ribavirin Concentrations Do Not Predict Sustained Virological Response in HIV/HCV-Coinfected Patients Treated with Ribavirin and Pegylated Interferon in the Swiss HIV Cohort Study

**DOI:** 10.1371/journal.pone.0133879

**Published:** 2015-07-28

**Authors:** Helen Kovari, Stefan Russmann, Bruno Ledergerber, Daniel Müller, Margalida Rotger, Pablo Velli, Matthias Cavassini, Juan Ambrosioni, Andrea Bregenzer, Marcel Stöckle, Enos Bernasconi, Andri Rauch, Roberto F. Speck, the Swiss HIV Cohort Study

**Affiliations:** 1 Division of Infectious Diseases and Hospital Epidemiology, University Hospital, University of Zurich, Zurich, Switzerland; 2 Department of Clinical Pharmacology and Toxicology, University Hospital Zurich, Zurich, Switzerland; 3 Institute of Clinical Chemistry, University Hospital Zurich, Zurich, Switzerland; 4 Institute of Microbiology, University Hospital, Lausanne, Switzerland; 5 Division of Infectious Diseases, University Hospital, Lausanne, Switzerland; 6 Division of Infectious Diseases, University Hospital, Geneva, Switzerland; 7 Division of Infectious Diseases, Cantonal Hospital, St. Gallen, Switzerland; 8 Division of Infectious Diseases and Hospital Epidemiology, University Hospital, Basle, Switzerland; 9 Ospedale Regionale, Lugano, Switzerland; 10 University Clinic of Infectious Diseases, University Hospital Berne and University of Berne, Berne, Switzerland; Emory University School of Medicine, UNITED STATES

## Abstract

**Background:**

Ribavirin (RBV) is an essential component of most current hepatitis C (HCV) treatment regimens and still standard of care in the combination with pegylated interferon (pegIFN) to treat chronic HCV in resource limited settings. Study results in HIV/HCV-coinfected patients are contradicting as to whether RBV concentration correlates with sustained virological response (SVR).

**Methods:**

We included 262 HCV treatment naïve HIV/HCV-coinfected Swiss HIV Cohort Study (SHCS) participants treated with RBV and pegIFN between 01.01.2001-01.01.2010, 134 with HCV genotype (GT) 1/4, and 128 with GT 2/3 infections. RBV levels were measured retrospectively in stored plasma samples obtained between HCV treatment week 4 and end of therapy. Uni- and multivariable logistic regression analyses were used to evaluate the association between RBV concentration and SVR in GT 1/4 and GT 2/3 infections. The analyses were repeated stratified by treatment phase (week 4-12, 13-24, >24) and *IL28B* genotype (CC versus CT/TT).

**Results:**

SVR rates were 35.1% in GT 1/4 and 70.3% in GT 2/3 infections. Overall, median RBV concentration was 2.0 mg/L in GT 1/4, and 1.9 mg/L in GT 2/3, and did not change significantly across treatment phases. Patients with SVR had similar RBV concentrations compared to patients without SVR in both HCV genotype groups. SVR was not associated with RBV levels ≥2.0 mg/L (GT 1/4, OR 1.19 [0.5-2.86]; GT 2/3, 1.94 [0.78-4.80]) and ≥2.5 mg/L (GT 1/4, 1.56 [0.64-3.84]; GT 2/3 2.72 [0.85-8.73]), regardless of treatment phase, and *IL28B* genotype.

**Conclusion:**

In HIV/HCV-coinfected patients treated with pegIFN/RBV, therapeutic drug monitoring of RBV concentrations does not enhance the chance of HCV cure, regardless of HCV genotype, treatment phase and *IL28B* genotype.

## Introduction

According to the World Health Organization, more than 185 million people globally have been infected with hepatitis C virus (HCV) [1]. In the last decade, the combination of ribavirin (RBV) and pegylated interferon (pegIFN) was the standard of care for treatment of chronic HCV infection. With the availability of direct-acting antivirals (DAAs), new treatment options with high cure rates for HIV/HCV-coinfected patients are available now. However, RBV is still a component of many of these regimens [2]. In addition, pegIFN/RBV remains standard of care in in resource limited settings because of financial constraints [1].

Hemolytic anemia is a common adverse effect of RBV, especially with higher dosages and prolonged therapy. RBV dose reduction and discontinuation are associated with reduced sustained virological response (SVR) rates [3]. Despite weight-adjusted dose regimens, *inter*individual RBV plasma concentrations vary widely [4, 5]. In contrast, *intra*individual variations are low and may be explained by the extensive volume of distribution and slow clearance from deep pharmacokinetic compartments [5, 6]. Steady-state concentrations are reached after 4 weeks [7]. Due to the large interindividual variability in RBV concentrations and a narrow therapeutic range, RBV therapeutic drug monitoring may be worthwhile to increase the likelihood of SVR while avoiding adverse events.

Some studies postulated that the level of RBV is a major determinant for SVR in the era of pegIFN/RBV in HIV/HCV-coinfected patients. However, this issue remains controversial (reviewed in [8]). In addition, data are inconsistent regarding the time point of plasma RBV concentration monitoring during treatment, and whether certain patient groups may benefit more than others. Therefore, we aimed here to assess the impact of RBV steady-state concentration on SVR at different treatment time points and in different HIV/HCV-coinfected patient groups treated with pegIFN/RBV in the Swiss HIV Cohort Study (SHCS).

## Methods

### Swiss HIV Cohort Study (SHCS)

The SHCS is an ongoing, prospective cohort study that continuously enrolls and observes HIV-infected adults at five university outpatient clinics, two large district hospitals, affiliated regional hospitals, and private practices, since 1988 [9]. Demographic, clinical and laboratory data are collected at registration and every six months thereafter using a standard protocol. The protocol was approved by local ethical review boards (Kantonale Ethik-Kommission Zürich, Kantonale Ethikkommission Bern, Ethikkommission beider Basel, Comite d’Ethique Geneve, Commission Cantonale d’Ethique Lausanne, Ethikkommission Kanton St.Gallen, Comitato etico cantonale Ticino), and written informed consent, including genetic analyses, was obtained from each patient. For this study, in addition to the information retrieved from the SHCS database, detailed information on HCV-infection, on treatment history and outcomes was obtained by retrospective structured and standardized chart review.

### Patient Selection

We included all HCV treatment naïve SHCS participants in the analyses with pegIFN/RBV therapy between 1 January 2001 and 1 January 2010, known HCV genotype (GT) and treatment response as well as availability of stored plasma samples from HCV treatment week 4 onwards for RBV concentration measurement. Patients with early therapies in acute HCV infections were excluded. None of the patients were on regimens containing DAAs. All patients received a standard regimen of weight-based RBV given twice a day and subcutaneous pegIFN. Due to the retrospective analysis of ribavirin plasma concentrations, the measured RBV concentrations did not have any influence on patients’ care.

### Laboratory Measurements and Genotyping

Blood samples were drawn semiannually at HIV cohort visits and stored at -80°C. RBV concentration was measured retrospectively in samples taken during HCV therapy between week 4 and the end of treatment. Plasma RBV concentrations were determined by high-performance liquid chromatography tandem mass spectrometry (LC-MS/MS) on a Finnigan TSQ 7000 (ThermoQuest, San Jose, USA) according to a validated and accredited method. It was shown before that RBV concentration remains stable under various storage conditions, including temperatures of -20°C and -80°C [10, 11]. Genotyping for *IL28B* SNP rs12979860 was done only in patients with available consent for genetic analyses. Genotyping was performed by using a custom TaqMan assay from Applied Biosystems as described by Ge et al [12].

### Definitions

HCV treatment outcome was defined as SVR, respectively non-SVR, including non-responders and relapsers, according to standard definitions [2]. SVR was defined as at least one negative HCV RNA test ≥12 weeks after the end of treatment [2]. A treatment course was considered terminated if pegIFN was discontinued for ≥30 days. Liver fibrosis stage was derived from liver biopsy using the METAVIR scoring system [13] or from transient elastography (Fibroscan, Echosens S.A.S.U., Paris, France), with a cutoff value of >12.5 kPa for Metavir F4 (Cirrhosis) [14]. Undetectable HIV RNA was defined as values <50 copies/mL.

### Statistical Analysis

A receiver operating characteristic (ROC) curve was used to determine the plasma RBV cut-off point that best discriminates between patients who achieved SVR and those who did not. Additionally, RBV plasma concentrations were dichotomized using the median RBV concentration of 2.0 mg/L in our cohort as the cutoff value. In addition, we performed also analyses with a higher cutoff value of 2.5 mg/L. For the overall treatment phase and for patients with more than one RBV concentration value per treatment phase we used the mean value. Median RBV values in the different treatment phases (week 4–12, 13–24, >24) were compared using the Wilcoxon rank-sum test. Since not all patients contributed samples for all treatment periods, we compared the demographic characteristics of the patients of the different treatment periods with chi-square and Kruskal-Wallis tests.

Univariable and multivariable logistic regression analyses were used to evaluate the association between RBV concentration levels and SVR. Analyses were done separately for HCV genotypes (GT) 1 or 4, and 2 or 3, respectively, because of the known different SVR rates for those two GT groups. Variables significantly associated with SVR in the univariable analyses and those considered to be clinically relevant were included in the multivariable model. Fixed covariables included sex, age, HIV transmission group, HCV RNA level, *IL28B* genotype, CD4 cell count, and antiretroviral therapy (ART) at HCV treatment start. Logistic regression analyses were further explored by using two different RBV concentration cutoff levels, and by stratifying by treatment phase and *IL28B* genotypes (CC versus CT/TT), respectively. All statistical analyses were performed using StataMP 13.1 (Stata Corp, College Station, USA).

## Results

### Study Population

**Fig 1:**
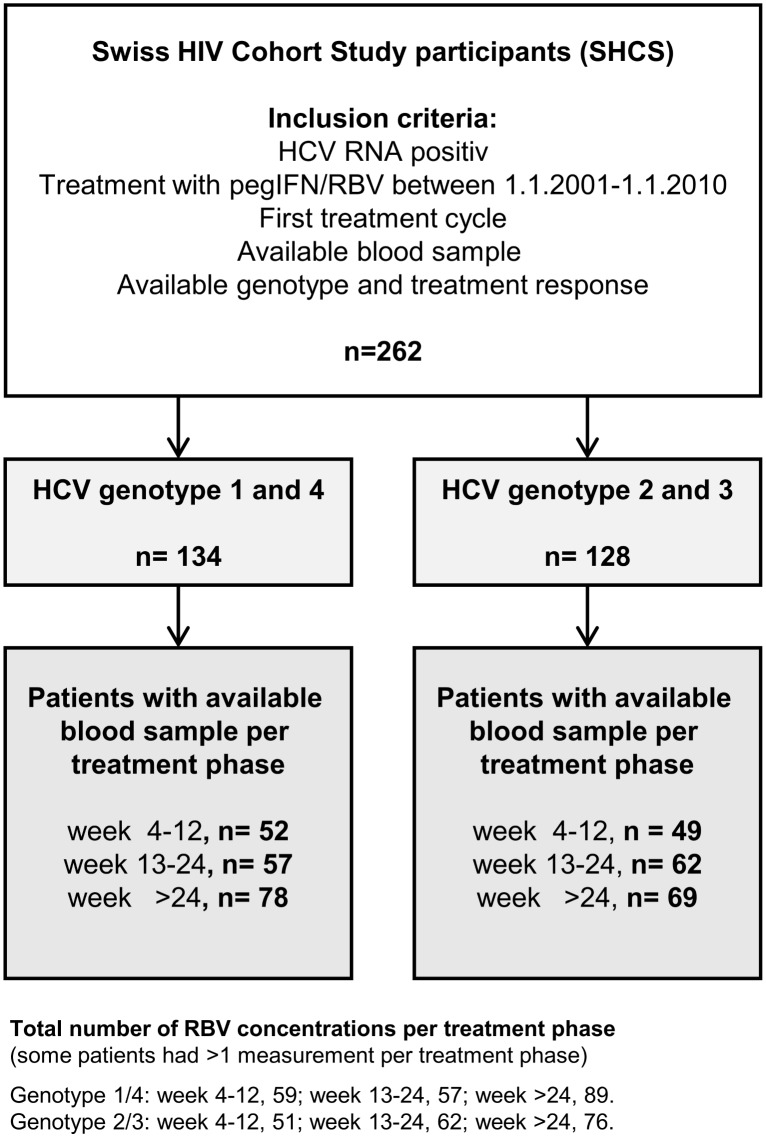
Patient flowchart.

We included 262 SHCS participants who fulfilled the inclusion criteria (Fig 1). Of those, 134 patients had HCV GT 1 or 4, and 128 GT 2 or 3 infections, respectively (Table 1). Among all included patients, the proportion of men was 72% and median age at HCV treatment start was 42 years. In 71% of the patients HIV was transmitted by intravenous drug use (IDU). Only 15/262 (6%) had CD4 cell counts less than 200 cells/μL and 81% were on antiretroviral therapy (ART) at HCV treatment start. Cirrhosis was present in 31% of participants. The *IL28B* CC genotype was documented in 51/116 (44%) persons with GT 1/4, and in 60/116 (52%) with GT 2/3 (in 30/262 patients genotyping was not performed because of missing genetic consent). SVR was achieved in 47/134 (35.1%) persons with GT 1/4, and in 90/128 (70.3%) with GT 2/3 infections. Baseline characteristics of patients stratified by HCV genotype (GT 1/4 vs.2/3) are shown in Table 1.

**Table 1:** Patient characteristics at hepatitis C virus (HCV) treatment start, treatment duration and sustained virologic response (SVR) according to HCV genotype group.

Variable		Genotype 1 and 4	Genotype 2 and 3
		n = 134	n = 128
Sex, n (%)	Male	98 (73.1)	90 (70.3)
	Female	36 (26.9)	38 (29.7)
Age, years, median (IQR)		42 (39–45)	42 (38–46)
Age, years	<45	90 (67.2)	85 (66.4)
	≥45	44 (32.8)	43 (33.6)
HIV transmission group (%)	MSM	16 (11.9)	8 (6.3)
	IDU	95 (70.9)	92 (71.9)
	Heterosexual	14 (10.5)	26 (20.3)
	Other	9 (6.7)	2 (1.6)
HCV RNA, available	n (%)	131 (97.8)	126 (98.4)
	<800’000 IU/ml	52 (38.8)	58 (45.3)
	≥800’000 IU/ml	79 (59.0)	68 (53.1)
Fibrosis score, available	n (%)	93 (69.4)	84 (65.6)
	Metavir <F4	62/93 (66.7)	61/84 (72.6)
	Metavir F4	31/93 (33.3)	23/84 (27.4)
*IL28B*, available	n (%)	116 (86.6)	116 (90.6)
	CC	51/116 (44.0)	60/116 (51.7)
	CT, TT	65/116 (56.0	56/116 (48.3)
CD4 cells/μl, (%)	<200	6 (4.5)	9 (7.0)
	200–349	19 (14.2)	29 (22.7)
	≥350	109 (81.3)	90 (69.5)
Not on ART		19 (14.2)	31 (23.4)
On AR	with HIV RNA undetectable	100 (74.6)	91 (71.1)
	with HIV RNA detectable	15 (11.2)	6 (4.7)
HCV treatment duration, weeks	median (IQR)	47.9 (27.6–50.4)	47.4 (26.4–49.0)
SVR, n (%)		47 (35.1)	90 (70.3)

Abbreviations: ART, antiretroviral therapy; HCV, hepatitis C virus; IDU, intravenous drug user; IQR, interquartile range; MSM, men who have sex with men; SVR, sustained viral response.

### RBV Plasma Concentration Levels

A total of 394 RBV drug levels were determined between week 4 and end of therapy. Number of patients with available RBV concentrations per genotype group and treatment phase is outlined in Fig 1. The patients contributing to the different treatment phases did not differ with regards to age, sex and transmission category (all p>0.06) besides patients from treatment phase week 4–12 versus 13–24 with GT 2/3 regarding transmission mode (p = 0.006) and age (p = 0.03). Overall median RBV plasma level was 2.0 mg/L in GT 1/4 and 1.9 mg/L in GT 2/3 infections (Table 2). Median RBV concentration was similar for all treatment phases (no significant difference between week 4–12, 13–24, and >24 in both genotype groups; all p-values >0.3). Since ROC analyses were not able to detect clearly discriminating cutoff values of RBV plasma concentration for the prediction of SVR, we dichotomized RBV levels as lower versus equal as or higher than the median (2.0 mg/L). In addition we also performed analyses using 2.5 mg/L as a cutoff (Table 2).

**Table 2:** Ribavirin plasma concentration overall and in different treatment phases.

Treatment phase		Overall		Week 4–12		Week 13–24		Week >24	
		GT 1/4	GT 2/3	GT 1/4	GT 2/3	GT1/4	GT 2/3	GT 1/4	GT 2/3
		n = 134	n = 128	n = 52	n = 49	n = 57	n = 62	n = 78	n = 69
RBV plasma level, median (IQR), mg/L		2.0 (1.5–2.8)	1.9 (1.5–2.5)	2.0 (1.5–2.8)	1.7 (1.5–2.4)	2.0 (1.5–2.7)	2.0 (1.5–2.4)	1.9 (1.4–2.8)	2.1 (1.5–2.7)
RBV plasma level, n (%)	<2 mg/L	67 (50.0)	67 (52.3)	26 (50.0)	30 (61.2)	27 (47.4)	31 (50.0)	41 (52.6)	30 (43.5)
	≥2 mg/L	67 (50.0)	61 (47.7)	26 (50.0)	19 (38.8)	30 (52.6)	31 (50.0)	37 (47.4)	39 (56.5)
RBV plasma level, n (%)	<2.5 mg/L	92 (68.7)	94 (73.4)	35 (67.3)	38 (77.6)	40 (70.2)	48 (77.4)	51 (65.4)	46 (66.7)
	≥2.5 mg/L	42 (31.3)	34 (26.6)	17 (32.7)	11 (22.5)	17 (29.8)	14 (22.6)	27 (34.6)	23 (33.3)

Abbreviations: GT, genotype; IQR, interquartile range; RBV, ribavirin.

### Impact of RBV Concentration on SVR

**Fig 2:**
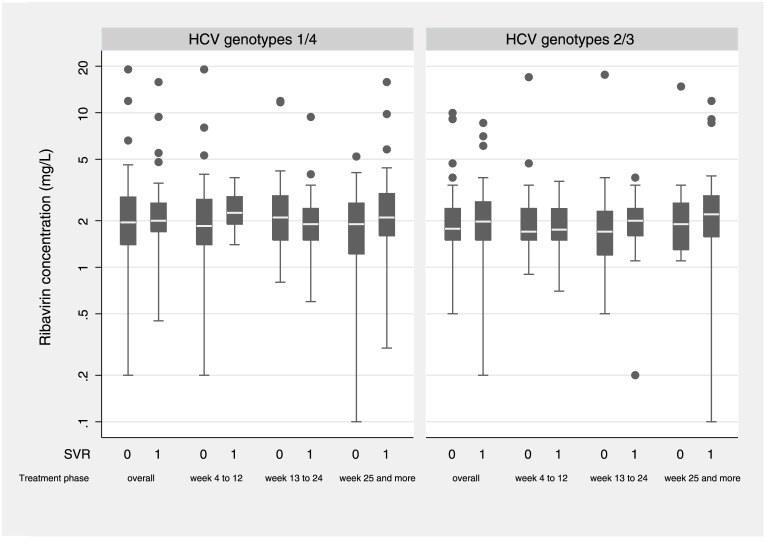
Comparison of ribavirin concentrations between HIV/HCV-coinfected patients with and without sustained virological response (SVR), stratified by HCV genotype and treatment phase. Abbreviations: SVR, sustained virological response; 0, no SVR; 1, SVR.

In patients with SVR RBV concentration was similar compared to patients without SVR, regardless of HCV genotype and treatment phase (Fig 2). RBV drug levels ≥2.0 mg/L and ≥2.5 mg/L were not significantly associated with SVR in both genotype groups (GT 1/4, ≥2.0 mg/L OR 1.19 [0.5–2.86]; ≥2.5 mg/L 1.56 [0.64–3.84]; GT 2/3, ≥2.0 mg/L 1.94 [0.78–4.80], ≥2.5 mg/L 2.72 [0.85–8.73]). In the multivariable regression analyses, *IL28B* genotype CC was a strong positive predictor for SVR in both genotype groups (GT 1/4, OR 3.96 [1.66–9.44], p = 0.002; GT 2/3, 3.02 [1.18–7.73], p = 0.02). A high HCV viral load was inversely correlated with HCV cure in GT 1/4 (0.38 [0.15–0.94], p = 0.04). Age, sex, and HIV transmission group had no significant influence on HCV treatment outcome (Table 3).

**Table 3:** Uni- and multivariable logistic regression analyses regarding factors associated with sustained virological response (SVR), stratified by HCV genotype group.

		Genotypes 1 and 4				Genotypes 2 and 3			
		n = 134				n = 128			
		Univariable Analysis		Multivariable Analysis		Univariable Analysis		Multivariable Analysis	
		OR (95% C.I.)	P-value	OR (95% C.I.)	P-value	OR (95% C.I.)	P-value	OR (95% C.I.)	P-value
Age, years	<45	1 (Ref.)		1 (Ref.)		1 (Ref.)		1 (Ref.)	
	≥45	0.59 (0.27–1.29)	0.1	0.46 (0.18–1.17)	0.1	1.14 (0.51–2.56)	0.8	1.12 (0.43–2.91)	0.8
Sex	male	1 (Ref.)		1 (Ref.)		1 (Ref.)		1 (Ref.)	
	female	0.63 (0.27–1.46)	0.3	0.40 (0.14–1.16)	0.09	2.33 (0.92–5.89)	0.08	1.96 (0.64–6.04)	0.2
HIV transmission group,	Non-IDU	1 (Ref.)		1 (Ref.)		1 (Ref.)		1 (Ref.)	
	IDU	0.52 (0.24–1.13)	0.1	0.56 (0.22–1.42)	0.2	0.88 (0.37–2.06)	0.8	0.83 (0.31–2.22)	0.7
HCV RNA, IU/ml	<800’000	1 (Ref.)		1 (Ref.)		1 (Ref.)		1 (Ref.)	
	≥800’000	0.42 (0.20–0.87)	0.02	0.38 (0.15–0.94)	0.04	0.78 (0.36–1.70)	0.5	0.76 (0.31–1.86)	0.5
*IL28B*	CT, TT	1 (Ref.)		1 (Ref.)		1 (Ref.)		1 (Ref.)	
	CC	3.19 (1.45–7.00)	0.004	3.96 (1.66–9.44)	0.002	2.67 (1.14–6.25)	0.02	3.02 (1.18–7.73)	0.02
CD4 cell count, cells/μl	<200	1 (Ref.)				1 (Ref.)			
	≥200	0.52 (0.10–2.70)	0.4			3.26 (0.82–12.88)	0.09		
Not on ART		1 (Ref.)				1 (Ref.)			
On ART		0.91 (0.33–2.51)	0.9			0.62 (0.24–1.60)	0.3		
RBV concentration, all FUP time	<2 mg/L	1 (Ref.)		1 (Ref.)		1 (Ref.)		1 (Ref.)	
	≥2 mg/L	1.27 (0.63–2.60)	0.5	1.19 (0.50–2.86)	0.7	1.29 (0.60–2.77)	0.5	1.94 (0.78–4.80)	0.2

Abbreviations: ART, antiretroviral therapy; C.I., confidence interval; FUP, follow-up; IDU, intravenous drug user; OR, odds ratio; RBV, ribavirin.

**Fig 3:**
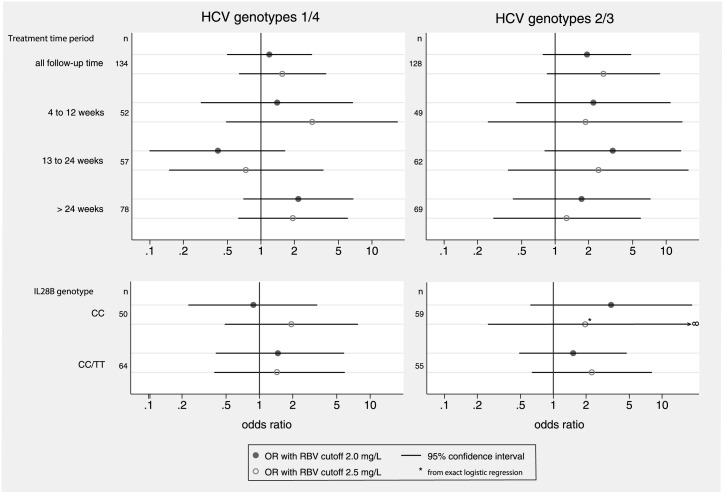
Impact of ribavirin level ≥2.0 mg/L and ≥2.5 mg/L on sustained virological response (SVR), stratified by HCV genotype group, treatment phase and *IL28B* genotype. Multivariable logistic regression analysis, adjusted for age, sex, HIV transmission group and HCV RNA level. Abbreviations: CI, confidence interval; HCV, hepatitis C virus; OR, odds ratio; RBV, ribavirin.

When we analyzed the association between RBV concentration and SVR in different treatment phases, neither RBV levels ≥2.0 mg/L, nor levels ≥2.5 mg/L in any of the treatment phases were significantly associated with HCV cure (Fig 3). When analyses were stratified by HCV genotype and *IL28B* genotype, RBV concentration levels remained without a significant impact on SVR in any group (Fig 3).

## Discussion

Several trials evaluated the impact of RBV therapeutic drug monitoring on SVR. Study results, however, remained controversial. We therefore assessed the correlation between RBV concentration and HCV cure in a large treatment naïve HIV/HCV-coinfected cohort treated with pegIFN/RBV. We found no difference in RBV concentrations between patients with and without SVR. RBV concentration levels ≥2.0 mg/L and ≥2.5 mg/L were not associated with HCV cure, regardless of HCV genotype, treatment phase (week 4–12, 13–24, >24), and *IL28B* genotype. Overall, median RBV concentrations were 2.0 mg/L in GT 1/4 and 1.9 mg/L in GT 2/3 infections, and there were no significant differences between treatment phases.

RBV concentrations ≥2.0 mg/L, as well as ≥2.5 mg/L, did not differentiate between patients responding to HCV therapy and those not. In line with our results, there are previous studies in mono- and coinfected patients that did not find an association between RBV concentration and virologic response [10, 15, 16]. However, there are also several other studies showing such a correlation. In coinfected patients, RBV cutoffs of 1.6 mg/L [17], 2.0 mg/L [18], 2.3 mg/L [6], and 2.5mg/L [19] were established to improve treatment efficacy. Reasons for the contradicting results may be small patient numbers, heterogeneity of populations and different clinical settings.

Previous trials showed that monitoring of RBV concentrations at weeks 4, 12, 24 (reviewed in [20]) or at treatment end [21] was associated with higher cure rates, while other studies only found a correlation of RBV concentration and increased response rates in difficult-to-treat patient groups, including GT 1 and 4 infections [6, 17], GT 1 coinfected patients with CT/TT *IL28B* genotypes [18], and African Americans with the CT/TT genotypes [22]. In our study, RBV concentrations at any treatment time point did not predict SVR. Moreover, we could not confirm that monitoring RBV steady-state levels may play a more important role in the achievement of HCV cure in patients with a higher risk of treatment failure.

Median RBV steady state concentrations of 2.0 mg/L in GT 1/4 and 1.9 mg/L in GT 2/3 in our cohort were similar as in other cohorts of HIV/HCV-coinfected [18] and HCV-monoinfected patients with values ranging from 1.4 to 2.5 mg/L [5, 21]. In a recently published study, RBV area under the curve (AUC_0-4h_) within the first hours after RBV intake was significantly lower in a small group of coinfected compared to monoinfected persons. The conclusion was that lower early bioavailability of RBV could be one of the reasons for lower SVR rates in coinfected patients [23]. Our results with similar RBV steady state concentrations in coinfected compared to monoinfected patients of other cohorts, do not support this hypothesis.

The overall SVR rates of 35.1% in GT 1/4 and 70.3% in GT 2/3 infections were high compared to other HIV/HCV-coinfected cohorts and similar to cure rates achieved in randomized controlled trials [24–26]. In a large cohort of US veterans, SVR was 16.7% in GT 1 and 44% in GT 2/3 infections during the same time period [27]. In our cohort, main predictors for SVR in both HCV genotype groups were *IL28B* genotype CC and low HCV RNA levels at baseline, which is in accordance with the results from other studies [19, 27, 28].

Major strengths of this study include the large number of patients and RBV concentration measurements in a nation-wide representative cohort of HIV-infected patients in a real-life setting. The large sample size was sufficient to allow separate analyses for GT1/4 and GT 2/3 HCV infections, and stratification by treatment phase and *IL28B* genotype. Limitations include the retrospective study design. We did not collect RBV trough plasma concentrations. However because of RBVs long elimination half-life and a stable steady-state after week 4, timing of sample collection is less relevant.

The new standard of care for HCV is DAA containing regimens, but RBV is still an essential backbone in many of these therapies. Our cohort does not yet provide information on the role of RBV drug monitoring in patients treated with DAA regimens. However, our data support current guidelines recommending weight-adapted RBV doses with close clinical monitoring and dose reduction, respectively discontinuation, in patients with signs of toxicity (dose-response relationship) rather than ribavirin concentration measurements (concentration-response relationship) [2], www.hcvguidelines.org). In resource limited settings where RBV/pegIFN is still standard of care and financial opportunities and laboratory infrastructure are limited our data does not support a role for RBV therapeutic drug monitoring [1].

In conclusion, we did not find a correlation between RBV plasma concentration and SVR, regardless of HCV genotype, treatment phase and *IL28B* genotype. Our data do not support RBV therapeutic drug monitoring in HIV/HCV-coinfected patients treated with pegIFN/RBV to enhance the chance of HCV cure.
